# Brain Radiation Necrosis: Current Management With a Focus on Non-small Cell Lung Cancer Patients

**DOI:** 10.3389/fonc.2018.00336

**Published:** 2018-09-05

**Authors:** Gokoulakrichenane Loganadane, Frédéric Dhermain, Guillaume Louvel, Paul Kauv, Eric Deutsch, Cécile Le Péchoux, Antonin Levy

**Affiliations:** ^1^Department of Radiation Oncology, AP-HP, CHU Henri Mondor, University of Paris-Est, Créteil, France; ^2^Department of Radiation Oncology, Gustave Roussy, Université Paris-Saclay, Villejuif, France; ^3^Department of Neuroradiology, AP-HP, CHU Henri Mondor, University of Paris-Est, Créteil, France; ^4^INSERM U1030, Molecular Radiotherapy, Gustave Roussy, Université Paris-Saclay, Villejuif, France; ^5^Université Paris-Sud, Université Paris-Saclay, Le Kremlin-Bicêtre, France

**Keywords:** complication, stereotactic radiotherapy, radiosurgery, vascular endothelial growth factor (VEGF), lung cancer, immunotherapy

## Abstract

As the prognosis of metastatic non-small cell lung cancer (NSCLC) patients is constantly improving with advances in systemic therapies (immune checkpoint blockers and new generation of targeted molecular compounds), more attention should be paid to the diagnosis and management of treatments-related long-term secondary effects. Brain metastases (BM) occur frequently in the natural history of NSCLC and stereotactic radiation therapy (SRT) is one of the main efficient local non-invasive therapeutic methods. However, SRT may have some disabling side effects. Brain radiation necrosis (RN) represents one of the main limiting toxicities, generally occurring from 6 months to several years after treatment. The diagnosis of RN itself may be quite challenging, as conventional imaging is frequently not able to differentiate RN from BM recurrence. Retrospective studies have suggested increased incidence rates of RN in NSCLC patients with oncogenic driver mutations [epidermal growth factor receptor (EGFR) mutated or anaplastic lymphoma kinase (ALK) positive] or receiving tyrosine kinase inhibitors. The risk of immune checkpoint inhibitors in contributing to RN remains controversial. Treatment modalities for RN have not been prospectively compared. Those include surveillance, corticosteroids, bevacizumab and local interventions (minimally invasive laser interstitial thermal ablation or surgery). The aim of this review is to describe and discuss possible RN management options in the light of the newly available literature, with a particular focus on NSCLC patients.

## Introduction

Due to its incidence and specific brain tropism, non-small cell lung cancer (NSCLC) represents the most common source of brain metastases (BM) (1). Given advances in systemic treatments with prolonged overall survival and better imaging [brain magnetic resonance imaging (MRI)] detection, BM incidence rate is increasing. The prognosis of BM NSCLC patients with targetable mutations has improved (2, 3), and recently available immune checkpoint blockers (ICI) provide promising prolonged outcome in non-mutated patients (4, 5). Altogether, up to 22% of NSCLC patients may have BM at the time of initial diagnosis, and BM will develop in approximately half of patients during their disease (6, 7). The BM rate may then be even higher in molecularly selected groups, such as epidermal growth factor receptor (EGFR) mutated or anaplastic lymphoma kinase (ALK) positive NSCLC patients (8).

The main focal treatment options for BM include surgery, stereotactic radiosurgery (SRT), and whole brain radiation therapy (WBRT). In the past decade, SRT has become the most frequently delivered focal treatment in patients with good prognosis criteria, and a limited number (<4) of BM (9–11). Frameless SRT delivers “ablative” dose, in a single or multiple course, as a definitive or postoperative treatment (12, 13). Focal high dose irradiation, as compared with neurosurgery, has the ability to treat inoperable sites, several lesions, and has the advantage to be less invasive. WBRT alone or in combination with SRT has been challenged in randomized trial, and its role is now limited to selected patients with multiple BMs ineligible for SRT (12, 14, 15). SRT is now often favored over WBRT due to a lower rate of adverse neurocognitive side effects. It has also been suggested that SRT without WBRT was feasible as the initial treatment for patients with 5–10 BMs (16). Local control at 1 year is generally high (88% in recent series), and SRT is generally considered as a cost-effective treatment (12, 17).

However, rare but potentially debilitating secondary late effects (3 months to several years post-irradiation) have been described after SRT. The most common delayed complication SRT is brain radiation necrosis (RN). RN may be particularly challenging in terms of diagnosis and treatment. Few studies have highlighted that RN may be more frequent in NSCLC patients harboring an oncogenic driver mutation. Within this review we aimed to describe and discuss the current knowledge regarding RN, with a special attention to NSCLC patients.

## Pathobiology

The physiopathology of radiation necrosis is still elusive and several hypotheses have been proposed. Implicated mechanisms in delayed RN include vascular injury, immune-mediated mechanisms and direct neural effects.

The vasculature damages are characterized by an increased permeability and a disruption of the blood brain barrier (BBB). High dose focal radiotherapy induces an endothelial cell loss through acid sphingomyelinase-dependent apoptosis (18) leading to vasogenic edema and ischemia. Tissue ischemia and vasogenic edema induce hypoxia, leading to reactive oxygen species production, affecting many cellular functions, and produce an increase of the hypoxia inducible factor (HIF-1α). HIF-1α subsequently upregulates the vascular endothelial growth factor (VEGF) secreted by astrocytes and endothelial cells. Immunohistochemistry of surgical samples of RN showed increased levels of VEGF in reactive astrocytes surrounding the core of necrotic tissue. VEGF exacerbates edema by increase of vascular permeability (19). These data indicate a crucial role of VEGF in the development and progression of RN and its inhibition could decrease the vascular permeability and therefore edema. Following these observation, anti-VEGF therapy has been one of the most tested compounds in the preclinical setting, and the sole pharmacological agent translating to clinical efficacy in the treatment of RN (cf. below, chapter on VEGF inhibition) (20, 21).

The immune system and peri-necrotic inflammation are also implicated in RN formation. Local infiltration of immune cells likely aggravates RN. VEGF induces the expression of adhesion proteins such as ICAM-1 on endothelial cells, and trigger pro-inflammatory cytokines [e.g.,: interleukin (IL)-1α, IL-6 and tumor necrosis alpha (TNF)-α] in animal models (18). Yoritsune et al. have also shown in human RN specimens, that astrocyte cells expressing the chemokine CXCL12 might attract CXCR4-expressing immune cells into the perinecrotic area, which in turn aggravates the local hypoxia (18, 22). The introduction of ICIs has significantly modified the therapeutic landscape of advanced NSCLC. As those agents are immunostimulatory, they could potentially exacerbate a preexisting inflammatory reaction in the context of RN.

Radiation induces white matter necrosis and oligodendrocytes demyelination. In the periphery of this necrotic zone, astrocytes, microglial cells and oligodendrocytes produce factors promoting cytokine release. A decrease of oligodendrocytes with incomplete neural stem cells or neuroblasts repopulation has been described (23, 24). Remyelinisation after human embryonic stem cell-derived oligodendrocyte progenitors transplantation is subsequently also assessed in preclinical models (25). Following these observations, many other agents than anti-VEGF have been tested in the experimental setting, but without reported favorable clinical effects (18).

## Clinical specificities of brain radionecrosis

The diagnosis of RN may be challenging. The main issue is to distinguish between RN and local recurrence (LR). When analyzing epidemiology or predictive factors of RN, one should keep in mind the possible subsequent bias related to diagnosis difficulties, as described below.

### Epidemiology and predictive factors

Reported clinical rate of RN is approximately 10%, with or without prior surgery (Table 1) (32, 35, 37). However, the rate of asymptomatic radiographical RN is higher: up to 25–30% in some series (29, 31). The cumulative incidence of RN is increasing over time after SRT. As an example, in a series from the Memorial Sloan Kettering, the actuarial incidence of RN was 5.2% at 6 months, 17.2% at 12 months, and 34% at 24 months (31). In another Japanese series, 16 patients with MRI contrast enhancement >18 months following SRT were identified. With a median follow-up of 48.2 months, 12 adverse radiation events (suspected radiological or pathological confirmed RN) occurred in a median follow-up of 33.2 months (38).

**Table 1:** Literature overview.

**Series**	**Date**	**N. of pts**	**N. of lesions**	**N. of NSCLC pts *N* (%)**	**NSCLC histology *N* (%)**	**Mutation status *N* (%)**	**median FU (mo.)**	**Incidence radiog. RN *N* (%)**	**Incidence symptom. RN *N* (%)**	**Risk factors RN**	**Trt RN**	**Efficacy**
Kim (26)	1997	77	91	77 (100)	ADK: 36 (47), SCC: 17 (22), LCC:13 (17), unclassified: 11 (14)	NM	8	NM	4 (4) lesions	NM	Surgery (*n* = 1)	NM
Saitoh (27)	2010	49	78	78 (100)	ADK: 36 (74), other: 13 (26)	NM	17.4	12.2 pts	6 (12) pts	NM	Steroids (*n* = 5), surgery (*n* = 1)	NM
Matsuyama (28)	2013	299	573	299 (100)	ADK: 210 (70), SCC: 34 (11), LCC: 5 (2), other: 10 (3), unknown: 40 (13)	NM	8,2		6 (2) pts	NM	HBOT (*n* = 6)	Improvement in all
Minniti (29)	2014	135	171	65 (48)	NM	NM	11.4	12 (9) pts	5 (6) pts	V18, V21	NM	NM
Won (30)	2015	64	123	64 (100)	ADK: 52 (82), SCC: 6 (9), LC: 4 (6), poorly diff. 2 (3)	NM	13.9	4 (6) pts	4 (6.1) pts	NM	Steroids (*n* = 3), surgery (*n* = 1)	NM
Kohutek (31)	2015	327	583	116 (43)	NM	NM	17.2	70 (26) lesions	47 (17) lesions	Maximal diameter	NM	NM
Miller (32)	2016	1939	5747	836 (43)	ADK: 530 (27), SCC: 97 (5), mixed/unknown: 209 (11)	EGFR+/ALK-: 35 (2), EGFR-/ALK+: 11 (1), EGFR-/ALK-: 104 (5)	12	427 (7) lesions	231 (4) lesions	Maximal diameter, heterogenity index	NM	NM
Ishihara (33)	2016	53	217	53 (100)	ADK: 29 (55), SCC:12 (23), others: 12 (23)	NM	8	20 (12) lesion)	6 (2.3) lesions	NM	Steroids (*n* = 6)	NM
Kim (34)	2017	1650	2843	699 (42)	NM	NM	NM	222 (8) lesions	120 (4) lesions	Targeted therapies (univ.) (anti VEGFR, anti EGFR, anti Her2), maximal diameter, heterogenity index	NM	NM
Keller^+^ (35)	2017	181	189	82 (45)	NM	NM	15	35 (19) pts	12 (7) pts	Infratentorial location	Surgery (*n* = 7)	NM
Martin (36)	2018	480	NM	294 (61)	NM	NM	23	48 (10) pts	48 (10) pts	ICI	Steroids (*n* = 18)	NM

*N. of, number of; NSCLC, non-small cell lung cancer; pts, patients; FU, follow-up; RN, radionecrosis; Trt, treatment; ADK, adenocarcinoma; SCC, squamous cell carcinoma; mo., months; symptom., symptomatic; radiog., radiographic; LC, large cell carcinoma; NM, not mentioned; diff, differentiated; univ., univariate; HBOT, hyperbaric oxygen therapy; ICI, immune checkpoint inhibitors; V18, V21, volume of brain receiving 18 and 21 Gy; Univ, univariate; heterogeneity index = maximum dose/prescribed dose*.

+ *Postop hypofractionated stereotactic radiation therapy only*.

Predictive risk factors associated with the development of RN cited in the literature link to BM, and treatment characteristics. Main accepted ones are a larger BM size, reirradiation, and higher total delivered radiotherapy dose (39, 40). Others criteria including BM features (location and deepness), radiotherapy parameters (high dose per fraction, volume of irradiated normal brain parenchyma [generally total volume of irradiated brain at a dose 12Gy or more]), and the use concurrent systemic therapy (including ICI) have been evocated but not systematically described (29, 41–45). In any case, fractionation (i.e., to increase the number of radiotherapy fractions), or the use of formulas for optimal individual SRT dose based on BM volume is encouraged to prevent RN (46).

Some authors advocated that RN occurrence might be more frequent in NSCLC patients (Table 1). In a NSCLC cohort of 836 patients (2,276 lesions), Miller et al. showed that lung adenocarcinoma histology (1-year incidence of 5.9% vs. 3.1–3.9% for other histologies), and ALK (HR 6.36, *p* < 0.001), but not EGFR lesions had increased rates of RN. The 1-year cumulative incidences of RN among EGFR+, ALK+, and ALK/EGFR wild-type lesions were 7.6, 17.3, and 3.7%, respectively. EGFR or ALK inhibitors, as compared to conventional treatments, were not associated with the occurrence of RN (32). Another series included 699/1,650 (42%) NSCLC patients who underwent SRS, with or without WBRT. Patients also received systemic treatments, including targeted therapies. NSCLC patients who received concurrent EGFR tyrosine kinase inhibitors (TKIs) had an increased of 12-month cumulative incidence of RN (15.6 vs. 6%, *p* = 0.04) as compared to other patients. This was more specifically observed in patients that received SRT+WBRT (*p* = 0.02) as compared with those receiving SRT without WBRT (*p* = 0.45) (34). It should anyway be emphasized that BM NSCLC patients with an oncogenic driver mutation generally receive more intensive local treatment, partly explaining the excess risk of toxicity (47).

The risk of ICI in contributing to RN is controversial. Prospective data is lacking and most retrospective series included melanoma patients (48). A retrospective SRT series reported a higher incidence of symptomatic RN for patients who received ICI as compared to those who did not. Among 480 patients with BM (289 [61%] of 480 NSCLC) who had been treated with SRT, 115 (24%) received an anti-PD1 (nivolumab or pembrolizumab) or an anti-cytotoxic T-lymphocyte associated protein 4 (ipilimumab). Patients treated with ICI had a significantly higher rate of symptomatic RN after adjustment for tumor type (HR: 2.6; *p* = 0.004). The risk of neurotoxicity was however highest for melanoma patients treated with ipilimumab (36). Other retrospective studies focusing on the outcome of patients with NSCLC with BM who received both cranial RT and an ICI did not report RN increase (49–52). However, it should be emphasized that pseudoprogression, observed with ICI may be difficult to be distinguished from RN or brain progression (53).

### Challenges in RN diagnosis

Radiographic changes (grade I, approximately 50%) from the symptomatic RN (grade II–IV) should be distinguished. In the latter case, an intervention may be required whereas a simple surveillance is sufficient for the former case. The symptoms depend on the location of the lesion, but manifest usually with focal neurologic signs and symptoms related to cerebral edema.

The main difficulty is to distinguish between RN and LR. Histology is the gold standard for a confirmed diagnostic. A recent series of BM patients who had a brain biopsy for RN or LR suspicion on MRI included 11/34 (31%) lung cancers patients. Most biopsies (24/35; 69%) showed RN only, and time from SRT to biopsy was significantly longer (>9 months) in the RN group (*p* = 0.004) as LR seemed to occur earlier than RN (54). On the other hand, brain biopsies are invasive and may not be accessible for all patients. Histopathologic interpretation of brain specimens could also be challenging due to heterogeneity of the lesion mixing irradiated residual tumor cells of indeterminate viability with RN that can be missed by the sampling, and some authors suggested that excision of the lesion only is able to determine its true histological nature (55).

**Figure 1:**
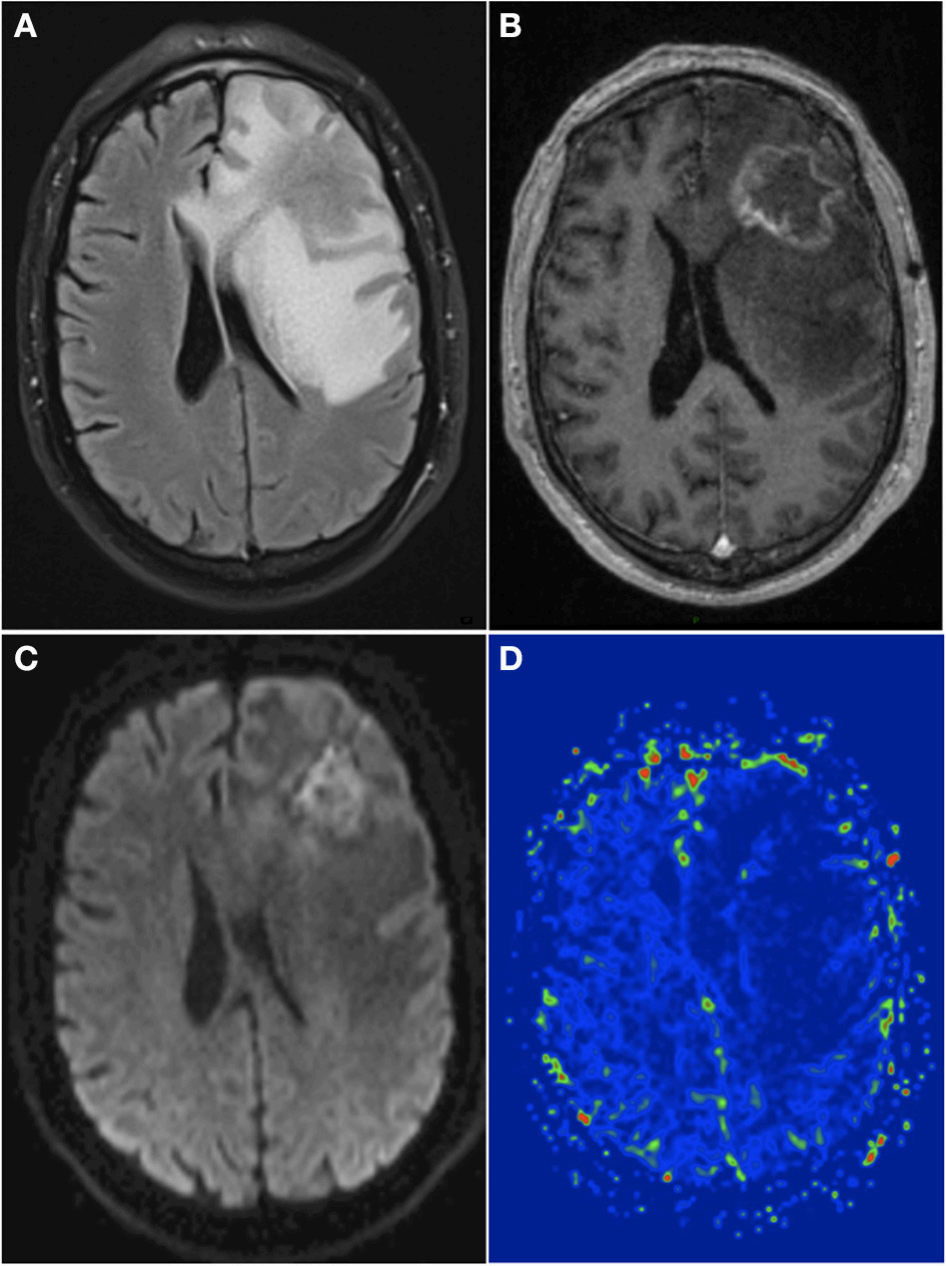
A 66-year-old man with history of brain metastasis of non-mutated NSCLC and treated by surgical resection and postop SRT. **(A)** Axial T2w FLAIR sequence showed a hyperintense signal appeared around the treated region 13 months after SRT. **(B)** T1w contrast sequence showed an inhomogeneous ring enhancement within the treated region. **(C)** DWI showed a low signal within the enhanced margin, with a high ADC (not shown). **(D)** Dynamic susceptibility contrast-enhanced perfusion weighted imaging showed a low hyperperfusion with a relative cerebral blood volume of 1.5, suggesting absence of tumor recurrence. Surgical resection confirmed the diagnosis of cerebral RN. NSCLC, non-small cell lung cancer; T2w, T2 weighted; T1w, T1 weighted; DWI, diffusion weighted imaging; ADC, apparent diffusion coefficient; SRT, stereotactic radiotherapy; RN, radionecrosis.

More often, non-invasive (clinical and radiographic) criteria are used, but the distinction between the RN and tumor can be particularly challenging. In most cases, conventional MRI shows a contrast-enhancing mass lesion with central necrosis and reactive edema contiguous to the site of the initial BM. “T1/T2 mismatch” (i.e., larger mass lesion seen in T2 sequence as compares with the T1 contrast-enhanced residual lesion) may favor RN (56). Dynamic (perfusion- and diffusion-weighted) MRI (Figure 1), and spectro-MRI (or magnetic resonance spectroscopy: MRS) have extensively been assessed to differentiate RN from LR. Dynamic susceptibility contrast-enhanced (DSCE) MR perfusion decreased parameters such as relative cerebral blood volume (rCBV) and relative peak height (rPH) or percentage of signal-intensity recovery (PSR) increase correlate with RN (57). On diffusion-weighted MR, decreased signal on diffusion-weighted imaging (DWI) and increased apparent diffusion coefficient (ADC) maps values reflect tumor control (58). MRS is an analytical technique that can be used to complement MRI in the characterization of tissue. Low lipid peak or high choline-to-creatine ratio and high choline-to-N-acetylaspartate (NAA) ratio on MR spectroscopy suggest tumor recurrence (59). Regarding positron emission tomography (PET), lower uptake with various radiotracers [fluorodeoxyglucose (FDG), methionine, fluorodihydroxyphénylalanine (Fdopa), fluoroéthyl-L-tyrosine, fluorocholine or thallium chloride-201 single-photon emission computed tomography (SPECT)] suggests necrosis. FDG has been the most commonly studied radiotracers but specificity is low, and the use of the couple dynamic MRI/PET is encouraged (60–64). Altogether, these imaging studies underline the difficulties to diagnose RN. Finally, the beneficial effect of steroids has also been incorporated to the diagnosis strategy, as depicted in the existing proposed algorithm to diagnose and treat RN (37, 64).

## Treatment options of radiation necrosis

RN can generally be managed conservatively without intervention. In symptomatic patients, moderate dose of glucocorticoids may produce prompt symptomatic improvement by reducing cerebral edema. Corticosteroids can then be gradually tapered. If not sufficient, RN management consists of VEGF inhibitors or laser interstitial thermal therapy (LITT). Ultimately, surgery may be required in patients who are resistant to other treatments, and/or to obtain a definitive diagnosis if a LR is suspected. Alternative approaches have been reported in some cases (therapeutic anticoagulation, antiplatelet therapy, and hyperbaric oxygen therapy), but may not be currently recommended.

### VEGF inhibition

As previously described, VEGF plays a critical role in the RN pathogenesis. Bevacizumab is the most commonly used anti-VEGF monoclonal antibody, and was prospectively evaluated in only one small prospective trial in the context of RN. Fourteen patients were randomized 1:1 to receive four cycles of intravenous (IV) bevacizumab at a dose of 7.5 mg/kg every 3 weeks vs. IV saline placebo. The primary endpoint was the change in edema volume on MRI (T2 FLAIR images) from baseline to the first evaluation at 6 weeks. Of note, there were no BM patients included but only prior irradiated primary central nervous system or head and neck tumors. Crossover was permitted, and the sample size was estimated to 16 patients. The 7 patients in the bevacizumab arm had a decreased volume of FLAIR edema with clinical amelioration whereas placebo arm patients demonstrated an increase in the volume of T2 weighted FLAIR edema (−59 vs. +14%, respectively; *p* = 0.01). Similarly, in patients receiving bevacizumab, a median decrease in the T1 weighted gadolinium enhancement (−63 vs. +17%; *p* = 0.006), and of the endothelial transfer constant (K-trans; a measure of capillary permeability in DCE MRI; −99 vs. +49%; *p* = 0.02) were reported. Six of 11 patients receiving bevacizumab had adverse events, with 3 serious adverse events: one aspiration pneumonitis, one pulmonary embolism secondary to deep vein thrombosis and one superior sagittal sinus thrombosis (65). Other retrospective series also reported a clinical benefit of bevacizumab, including reduction in steroid requirement (66–68).

Those promising results should nevertheless be tempered. One should not forget that bevacizumab has a certain activity on BM in NSCLC patients, especially when we know that LR and RN can be associated in a significant proportion of cases (69). Development of RN was also observed among 24/271 (9%) patients receiving SRT with concurrent bevacizumab (31). Worsening of symptoms may occur, and RN recurrences after bevacizumab withdrawal have been described (70). In a series including a majority (11/14; 79%) of BM from primary lung cancer, clinical improvement was seen in 13/14 cases (92.9%), but the 10/13 responsive patients (76.9%) exhibited a recurrence of brain necrosis after bevacizumab discontinuation (71). Bevacizumab is a promising treatment option for RN, but needs to be validated in larger prospective studies.

### Invasive interventions

LITT is a stereotactic-guided minimally invasive ablative technique that generates high temperature, resulting in tissue coagulation necrosis, angiogenesis eradication, and cellular apoptosis. The use of LITT-guided MRI allows to control accurately the delivery, and to spare the surrounding healthy tissues. LITT has been used in several situations in neurology, including RN. Most of the available data come from small retrospective studies. Rao et al. reported the results of MRI-guided LITT for 12/15 (80%) NSCLC patients with suspected RN or LR after SRT for BM. On average, the lesion size measured 3.7 cm. Authors were able to perform 3.3 ablations per treatment, in a total ablation time of 7.5 min. The local control was high (76%) at a median follow-up of 6 months, with two patients experiencing recurrence at 6 and 18 weeks after the procedure (72). The largest series, from the University of Arizona, consisted of 25 patients with suspected RN, occurring after treatment for 18 primary brain tumors and 7 BM. Progression free and overall survival rates in patients with BM were 11.4 and 55.9 months, respectively. The quality of life analysis showed an improvement on mental health and vitality at 12 months (73). One of the advantages of this technique is the possibility to perform a biopsy prior to treatment to confirm the diagnosis of RN. Moreover, LITT is a reasonable option in case of LR, considering the efficacy and secondary effects of other treatment modalities (i.e., reirradiation).

Surgery allows pathological confirmation, and the rapid relief from mass effect and brain edema. In a series of 15 patients with RN, the surgery improved the neurological symptoms in 14 cases. Pure RN was histologically determined for 50% of operated patients. In the algorithm proposed by the authors, patients with significant increased edema volume with mass effect, or becoming symptomatic despite steroids trial should undergo surgery (37). Another surgical series for patients with RN reported that 9 had a steroid dose reduction, 4 improved their performance status score (4 stable and 3 deterioration), and neurologic deficits were ameliorated in 4 (4 stable). Nonetheless, 2 worsened their neurologic deficit and one patient developed a new neurologic deficit after surgery. This study highlights the potential morbidity of surgical resections of RN, and suggests reserving surgery for symptomatic patients in whom medical treatment has failed (74).

## Perspectives and concluding remarks

Newer generation TKIs will possibly modify the therapeutic sequences in advanced mutated NSCLC patients. In retrospective studies, the deferral of radiation therapy (SRT or WBRT) was usually associated with inferior survival rates in oncogenic driver mutation patients (75, 76). However newer generation TKI such as first-line alectinib (*ALK*+ patients) and osimertinib (*EGFR* mutated patients) provided superior intracranial control compared to standard of care (2, 3). This, with the increased use of ICI, may then possibly lead to a decreased use of SRT, and subsequently change the RN rate occurrences in NSCLC patients. Moreover, NSCLC mutated patients have potentially an increased incidence of RN due to tumor biology or the use of concurrent TKI, but this remains to be confirmed.

An ongoing randomized phase II trial (BeSt Trial;Alliance A221208; NCT02490878) from the MD Anderson is investigating whether the addition of bevacizumab (10 mg/kg IV at day one and 15 for four cycles) to standard corticosteroid therapy could result in greater improvement of RN symptoms (primary endpoint: patient-reported outcome of RN up to 8 weeks). One hundred thirty patients should be included and eligibility criteria encompass perfusion-imaging parameters of RN susceptibility (high PSR and low rCBV). Another multicenter prospective French trial (CV-METANEC;NCT02636634) has recently been completed. It compared PET-FET (1-Fluoro-Ethyl-Tyrosine) and magnetic resonance spectroscopy to histological results in patients receiving brain biopsy for active persistent and increased brain lesion 4 months after SRT. The results of such studies should help to differentiate RN from LR after SRT, and help to guide clinicians to select an appropriate treatment for patients.

## Author contributions

GL and AL designed and outlined structure and contents of the review. All authors contributed to the literature analysis, interpretation, and writing of the review.

### Conflict of interest statement

The authors declare that the research was conducted in the absence of any commercial or financial relationships that could be construed as a potential conflict of interest.
